# Divergent Roles for the IL-1 Family in Gastrointestinal Homeostasis and Inflammation

**DOI:** 10.3389/fimmu.2019.01266

**Published:** 2019-06-07

**Authors:** Craig P. McEntee, Conor M. Finlay, Ed C. Lavelle

**Affiliations:** ^1^Faculty of Biology, Medicine and Health, School of Biological Sciences, Lydia Becker Institute of Immunology and Inflammation, University of Manchester, Manchester, United Kingdom; ^2^Faculty of Biology, Medicine and Health, Manchester Collaborative Centre for Inflammation Research, School of Biological Sciences, University of Manchester, Manchester, United Kingdom; ^3^Faculty of Biology, Medicine and Health, Wellcome Trust Centre for Cell-Matrix Research, School of Biological Sciences, University of Manchester, Manchester, United Kingdom; ^4^Adjuvant Research Group, School of Biochemistry and Immunology, Trinity Biomedical Sciences Institute, Trinity College Dublin, Dublin, Ireland; ^5^Centre for Research on Adaptive Nanostructures and Nanodevices (CRANN), Advanced Materials and BioEngineering Research (AMBER), Trinity College Dublin, Dublin, Ireland

**Keywords:** cytokine, inflammation immunomodulation, gastrointestinal, interleukin-1, inflammatory bowel conditions

## Abstract

Inflammatory disorders of the gastro-intestinal tract are a major cause of morbidity and significant burden from a health and economic perspective in industrialized countries. While the incidence of such conditions has a strong environmental component, in particular dietary composition, epidemiological studies have identified specific hereditary mutations which result in disequilibrium between pro- and anti-inflammatory factors. The IL-1 super-family of cytokines and receptors is highly pleiotropic and plays a fundamental role in the pathogenesis of several auto-inflammatory conditions including rheumatoid arthritis, multiple sclerosis and psoriasis. However, the role of this super-family in the etiology of inflammatory bowel diseases remains incompletely resolved despite extensive research. Herein, we highlight the currently accepted paradigms as they pertain to specific IL-1 family members and focus on some recently described non-classical roles for these pathways in the gastrointestinal tract. Finally, we address some of the shortcomings and sources of variance in the field which to date have yielded several conflicting results from similar studies and discuss the potential effect of these factors on data interpretation.

## Tolerogenic Mechanisms of the Gastrointestinal Tract:

**Figure 1:**
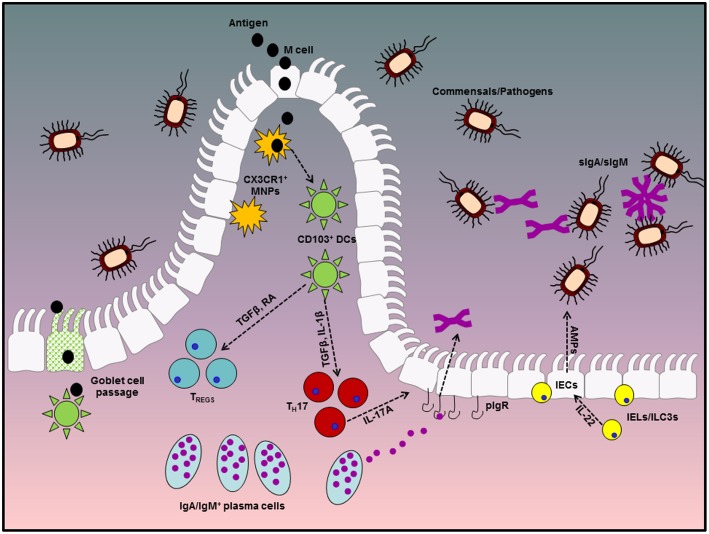
Diagrammatic representation of tolerogenic mechanisms in the gastrointestinal tract. Homeostasis at the gastrointestinal mucosa involves several specialized immune cells and cross-talk with symbiotic microbes in the lumen. The first line of defense is provided by antibodies in the gut lumen, which are transported across the epithelial barrier via the pIgR, in conjunction with IEC-derived AMPs, production of which is stimulated by IEL or ILC-derived IL-22. Beneath the epithelial layer, the intestinal lamina propria is home to mononuclear phagocytes which can capture antigen either directly from the lumen via trans-epithelial extension of dendrites or through M cell passages. These MNPs can in turn pass antigenic material to migratory CD103^+^ DCs which favor a tolerogenic immune response through their ability to metabolize vitamin A into retinoic acid and activate latent TGFβ, both of which are involved in the differentiation of T_REGS_ and IgA class switch recombination in B cells. T_REGS_ and IgA-secreting plasma cells play fundamental roles in remaining tolerant to innocuous antigens from diet and the microbiota and dysregulated responses in either of these factors significantly enhances susceptibility to enteric inflammatory disorders.

The gastrointestinal tract (GIT) is the largest internal organ in the human body with an estimated surface area in excess of 400 m^2^ and has evolved to meet several physiological and biological needs. However, in order to meet these demands, part of the evolutionary process of the GIT has been to facilitate colonization by a vast repertoire of mutually beneficial micro-organisms which aid in the metabolism of certain dietary components and are of fundamental importance to the overall well-being of the host. These microbes, which include fungi, bacteria, viruses, and bacteriophages, are collectively referred to as the commensal microbiota or microflora and together with the metabolites they produce form the host microbiome. In addition to aiding in the breakdown and absorption of dietary factors, these microbes also actively compete with enteric pathogens for essential nutrients and environmental niches and facilitate the development and maturation of a robust mucosal immune repertoire. Indeed studies with germ-free mice have highlighted that the lack of a commensal microbiota in these animals results in significant barrier defects and enhanced intestinal permeability. However, given their constant presence and the enormity of the antigenic burden faced by the GIT, robust mechanisms have evolved to prevent aberrant effector immune responses to these innocuous antigens (Figure 1).

While distinct regions of the GIT differ in terms of their anatomical, physiological and immunological make-up, consistent throughout is the presence of a single layer of columnar intestinal epithelial cells (IECs) which act as a physical blockade between the luminal compartment and the typically sterile underlying sub-epithelial layers (1). In an effort to preserve homeostasis, these IECs are typically hypo-responsive to innate stimuli such as the bacterial pathogen-associated molecular pattern (PAMP) lipopolysaccharide (LPS) (2, 3). This is achieved in part by the compartmentalization and spatio-temporal organization of various pathogen recognition receptors (PRRs), including several toll-like receptors (TLRs), in these cells. Indeed the expression of several TLRs is of restricted to the baso-lateral cell membrane IECs, away from the plethora of antigens to which the apical surface of these cells is exposed.

The term “brush border” has been used to describe the physical appearance of the small intestinal epithelium as the columnar enterocytes have protrusions called microvilli on their apical surface. These microvilli not only aid in physiological processes such as peristalsis and mucous sloughing, but also provide additional surface area for the absorption of dietary nutrients. Furthermore, these villous protrusions harbor digestive enzymes and transporter channels which are essential for host metabolism and osmotic homeostasis. Overlying the microvilli is a thick glycocalyx consisting of mucins and other glycoproteins which helps to prevent colonization of the intestinal epithelium by commensal bacteria and potential pathogens alike. Under steady-state conditions in a healthy GIT, adjacent IECs are closely associated to one another by tight junction proteins, thereby preventing the passive transfer of antigen-bearing material from the microbe-rich lumen into the comparatively sterile sub-epithelial layer. As a result, the sampling of luminal contents does not occur randomly but is instead primarily restricted to highly specialized microfold or M cells, so called due to their unique morphological features which includes short, loosely packed microvilli and a lack of surface glycocalyx. These cells are located in the follicle associated epithelium (FAE) which overlies lymphoid structures known as Peyer's patches (PPs), one of the primary immune-inductive sites of the gut-associated lymphoid tissue (GALT). Their differentiation and functional maturation from leucine-rich repeat containing G protein-coupled receptor positive (Lgr^+^) stem cells is driven by the receptor activator of NF-κB ligand (RANKL), expression of which is typically restricted to stromal cells situated directly beneath the FAE. Signaling of RANKL through its receptor RANK, which is expressed by all epithelial cells, induces the functional maturation of M cells through up-regulation of the transcription factor Spi-B (4, 5). As a result, somewhere in the region of 10% of epithelial cells of the murine FAE can be identified as M cells at steady state (6). In addition to M cells, a certain degree of antigen sampling has also been reported to occur via goblet cell passages as well as directly from the lumen by CX3CR1^+^ mononuclear phagocytes which have been shown to extend their dendrites through epithelial tight junctions without compromising barrier integrity (7–10).

As one of the primary portals of entry for potentially harmful pathogens, the immune system of the GIT must be capable of responding rapidly in the event of a barrier breach. Integral to this initial response are a number of T cell subsets which reside within, or in close proximity to, the epithelial layer and are therefore referred to as intra-epithelial lymphocytes (IELs). These IELs can be broadly sub-divided into two main groups, conventional T cell receptor (TCR) αβ cluster of differentiation 4 positive (CD4+) or CD8αβ-expressing cells (TCRαβ^+^ CD4^+^ or TCRαβ^+^ CD8αβ^+^ respectively), or unconventional T cells expressing an invariant heterodimeric TCR consisting of gamma and delta chains - γδ T cells - or TCRαβ^+^ cells expressing a homodimeric CD8αα co-receptor, both of which have a divergent ontogeny to conventional IELs (11). In addition to these IELs, the GIT harbors an abundance of other atypical lymphoid cells which are primed to respond rapidly to various stimuli and include lineage negative (Lin^−^) innate lymphoid cells (ILCs), as well as non-classical major histocompatibility complex (MHC)-restricted invariant natural killer T (iNKT) cells and mucosal-associated invariant T (MAIT) cells, which recognize antigens presented by cluster of differentiation 1d (CD1d) or major histocompatibility complex-related molecule 1 (MR1) (12–15). Collectively, these cells serve as the first responders to microbial colonization or epithelial damage by rapidly secreting cytokines which in turn stimulate the production of anti-microbial peptides (AMPs) or bolster epithelial integrity (16–18). However, in order to prevent aberrant effector immune responses to the plethora of innocuous stimuli to which the GIT is constitutively exposed, it is essential that all of the aforementioned processes are tightly regulated so that immune quiescence can prevail under steady-state conditions. Furthermore, the lamina propria (LP) of the GIT is populated by highly specialized tolerogenic dendritic cells (DCs), which preferentially drive the differentiation of anti-inflammatory regulatory T cells (T_REGS_) via their expression of vitamin A metabolizing enzymes and transforming growth factor beta (TGFβ)-activating integrins (19–23). Disruption in one or more of these aforementioned tolerogenic mechanisms can result in a number of gastrointestinal disorders including, but not limited to, bacterial overgrowth, food intolerance and/or inflammatory bowel disease (IBD). The latter of these conditions consists clinically of Crohn's Disease (CD) and Ulcerative Colitis (UC), two distinct afflictions both characterized by chronic relapsing and remitting inflammation of the entire GIT or colon, respectively. Recent reports have identified particularly high incidences of IBD in Europe and North America, with an estimated incidence of 300–500 per 100,000 individuals (24). The identification of environmental or genetic factors correlating with disease susceptibility and the elucidation of the exact mechanisms responsible for disease pathogenesis is essential for the future development of efficacious therapies.

The interleukin (IL)-1 family of cytokines and receptors have been extensively studied in the context of IBD as a result of their known role in the etiology of several inflammatory disorders. Therapeutic targeting of these pathways has been investigated for the amelioration of IBD symptoms, with variable results observed to date. Here we shall discuss the current literature as it pertains to the IL-1 family and its dichotomous roles as both a regulator of intestinal immune homeostasis and driver of inflammatory responses.

## The IL-1 Family

**Figure 2:**
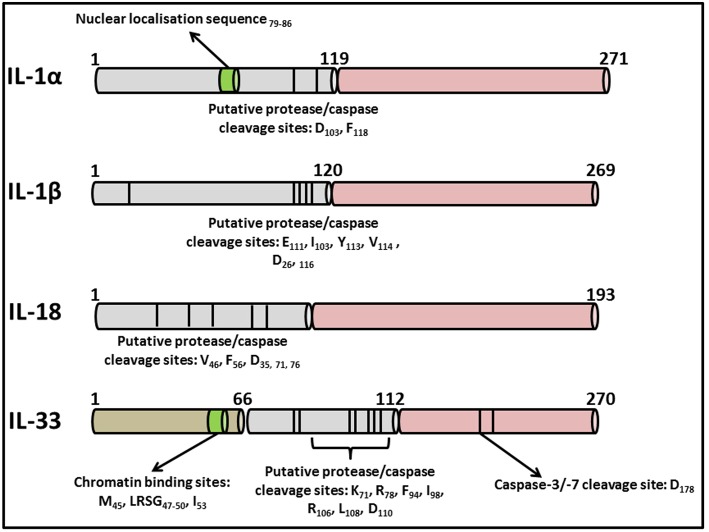
Primary structures of IL-1 family cytokines. Diagrammatic representation of the primary structures of well characterized cytokines belonging to the IL-1 superfamily. While all contain consensus cleavage sites for caspases and/or inflammatory proteases, IL-1α and IL-33 also contain nuclear localization sequences in their N-terminal domains enabling them to traffic to the nucleus from where they can regulate inflammatory gene transcription. Activation of these cytokines via post-translational cleavage events has been extensively reviewed elsewhere (38).

The IL-1 family consists of 11 distinct members comprising the immunomodulatory cytokines, IL-1α, IL-1β, IL-18, IL-36α, IL-36β, IL-36γ, and IL-33 in addition to four natural antagonists—IL-1Ra, IL-36Ra, IL-37, and IL-38 as outlined in Table 1. Several of the family members exhibit remarkable sequence homology to one another and contain several conserved primary structural features as outlined in Figure 2. Indeed the genes encoding almost all of these cytokines, with the exception of *IL33* and *IL18*, cluster to a small section of chromosome 2 in humans (25). IL-1 family cytokines are produced by a vast repertoire of immune cells including monocytes, DCs, macrophages, natural killer (NK) cells, activated T and B cells, as well as non-hematopoietic cells including epithelial cells and keratinocytes in response to pathogen- and damage-associated molecular patterns (PAMPs and DAMPs), as well as other cytokines, in particular Tumor Necrosis Factor (TNF) (26).

**Table 1:** Summary of the IL-1 family of cytokines in humans.

**Cytokine:**	**Receptor**	**Accessory protein**	**Antagonist**	**Source**	**Targets**
IL-1α/β (*IL1A/IL1B* or *IL1F1/IL1F2*)	IL-1RI (*IL1R1*)	IL-1RAcP (*IL1RAP*)	IL-1Ra (*IL1F3*), IL-1RII (*IL1R2*), sIL-1RII, SIGIRR (*SIGIRR*)	IECs, DCs, mø, CD4^+^ T cells	DCs, stem cells, neutrophils, ILC3s, mø, monocytes, TCRαβ, TCRγδ, B cells
IL-18 (*IL18* or *IL1F4*)	IL-18Rα (*IL18R1*)	IL-18Rβ (*IL18RAP*)	IL-18BP (*IL18BP*)	IECs, mø, DCs	Neutrophils, mø, NK, endothelial, smooth muscle, T and B cells
IL-33 (*IL33* or *IL1F11*)	ST2 (*IL1RL1*)	IL-1RAcP (*IL1RAP*)	sST2	IECs, mast cells, DCs, endothelial cells, astrocytes, cardiomyocytes	ILC2s, CD4^+^, CD8^+^ T cells, keratinocytes
IL-36α, β, γ (*IL36A/B/G* or *IL1F6/IL1F8/IL1F9*)	IL-36R (*IL1RL2*)	IL-1RAcP (*IL1RAP*)	IL-36Ra (*IL1F5*), IL-38	Epithelial cells, keratinocytes, DCs, mø	Keratinocytes, monocytes, DCs, CD4^+^ T cells
IL-37 (*IL37* or *IL1F7*)	IL-18Rα (*IL18R1*)	SIGIRR (*SIGIRR*)	N/A	NK cells, monocytes, B cells	DCs, T cells, endothelial cells
IL-38 (*IL1F10*)	IL-36R (*IL1RL2*)	IL-1RAcP (*IL1RAP*)	N/A	Apoptotic mø, cancerous cells	T cells, endothelial cells, mø

**Figure 3:**
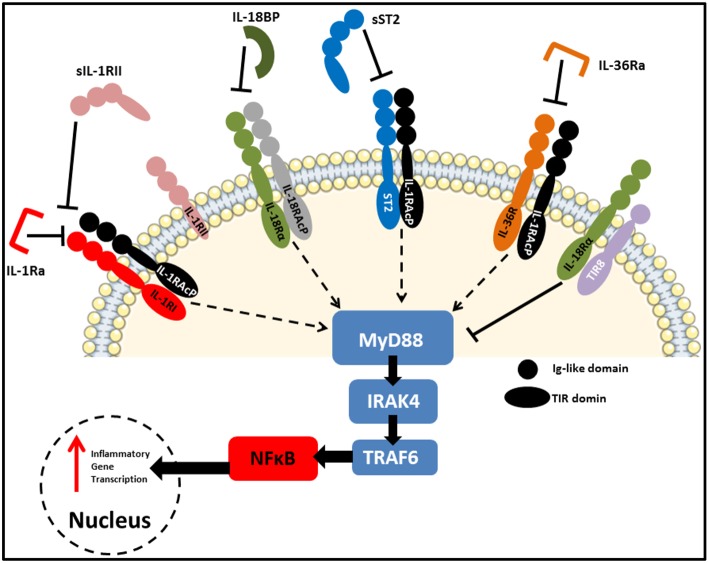
IL-1 family receptors and negative regulators. Receptors of the IL-1 family bear close resemblance to TLRs in that they consist of an extracellular ligand-binding domain, a transmembrane helix and an intracellular TIR domain which mediates MyD88-dependent signal transduction. Due to the highly pleiotropic nature of IL-1 family cytokines, several natural antagonists and decoy receptors have been identified which ensure tight regulation of their functions. These inhibitory molecules can target the receptors themselves, as is the case for IL-1Ra and IL36Ra, or the active cytokines to prevent them from binding to their cognate receptors. Examples of the later include IL-18BP, as well as soluble and membrane-bound IL-1RII and soluble ST2. Finally, SIGIRR (TIR8) acts as an inhibitory receptor by sequestering intracellular kinases required for signaling via engaged IL-1 family receptors.

As reviewed extensively elsewhere, receptors of the IL-1 family share remarkable structural similarity to those of the Toll-like receptors (TLRs). As a result, these two families are often grouped together as the Toll/IL-1 Receptor (TIR) superfamily (27). Figure 3 highlights some of these similarities, with each receptor consisting of an extracellular ligand-binding domain, a transmembrane helix and an intracellular TIR domain which is essential for signal transduction. Additionally, several members of both families use myeloid differentiation primary response 88 (MyD88) as an adaptor protein for downstream signal propagation and they all utilize the IL-1 receptor accessory protein (IL-1RAcP). Specific sequence residues in the extracellular immunoglobulin-like domain of IL-1 family receptors confers specificity for their cognate ligands (28). The outcome of signaling through several IL-1 receptors (IL-1Rs) is similar to MyD88-dependent TLR signaling, due to the significant homology between the IL-1R and the TLRs which both contain cytosolic TIR domains capable of interacting with MyD88 (29). The binding of IL-1 to the type I IL-1R (IL-1R1), enables the receptor complex to recruit and bind to IL-1RAcp (30). This dimerization event is essential for the subsequent transduction of the IL-1 signal downstream, culminating in the expression of several IL-1-dependent pro-inflammatory cytokines (31, 32). Furthermore, signaling molecules such as the IL-1R-associated kinases (IRAKs) and TNF receptor-associated factor 6 (TRAF6), also present in many of the TLR signaling pathways, are also essential for IL-1 signaling. Therefore, it is not surprising that signaling through both TLRs and the IL-1R culminates in the expression of genes encoding pro-inflammatory cytokines and chemokines through activation of nuclear factor kappa B (NFκB) and mitogen-activated protein (MAP) kinases.

Signaling through the IL-1R transduces a multitude of signals many of which are capable of modulating both local and systemic immune responses. IL-1β is a highly pleiotropic cytokine and prior to the introduction of the interleukin nomenclature, IL-1β was given the name leukocyte activating factor (LAF) due to its broad range of downstream effects. Not only is it a key cytokine involved in both chronic and acute inflammation (33, 34), it is also capable of increasing the expression of adhesion molecules on endothelial cells, inducing the functional maturation of DCs, driving the recruitment and activation of lymphocytes and NK cells while also serving as an endogenous pyrogen with the potential to cause fever (26). In addition to these classical pro-inflammatory functions, several IL-1 family cytokines are capable of modulating downstream effector T cell responses by directing their differentiation down different pathways. For example, IL-1β is known to drive the differentiation of T_H_17 cells whereas IL-18 and IL-33 favor the expansion of T_H_1 and T_H_2 cells, respectively (35, 36). Additionally, these cytokines can also elicit stimulate the production of cytokines from various ILCs and IELs which constitutively express receptors for IL-1 family cytokines. IL-1β is capable of driving robust IL-17 secretion from both γδ T cells and type 3 ILCs (ILC3) whereas IL-18 and IL-33 can induce the rapid secretion of type 1 or type 2 cytokines from ILC1s and ILC2s, respectively (37). Owing to these pro-inflammatory capabilities, together with the diverse nature of cells expressing one or more IL-1 family receptors, the activities of IL-1 family cytokines are tightly regulated and all, apart from IL-1Ra, are initially synthesized as biologically inactive precursors which require post-translational modifications before they can signal via their cognate receptors. Maturation of IL-1β, IL-18, and IL-37 requires their pro-forms to be cleaved by inflammatory caspases while post-translational cleavage events are also a feature of IL-1α, IL-33 and the IL-36 sub-family (38). Additionally, the active cytokines themselves are endogenously regulated by a network of naturally occurring antagonists and/or decoy receptors capable of blocking pro-inflammatory signal transduction by IL-1 family members, thereby providing an additional layer of regulation governing the activities of this pleiotropic family of cytokines (39).

The IL-1 family cytokines are typically associated with pro-inflammatory responses and IL-1β has been identified as a key mediator of inflammation-induced pathology in several autoimmune and auto-inflammatory conditions including experimental autoimmune encephalomyelitis (EAE)—the murine model of multiple sclerosis (MS)—and obesity-associated airway hyper-reactivity (40–42). As a result, these cytokine pathways have emerged as candidate targets for several conditions, including IBD (43). Indeed therapeutic blockade of the IL-1R via subcutaneous administration of a recombinant IL-1Ra—anakinra (Kineret®)—is licensed for the treatment of rheumatoid arthritis (RA) in patients who fail to respond to anti-TNF and in individuals afflicted by cryopyrin-associated periodic syndromes (CAPS) (44). Moreover, canakinumab (Ilaris®), is a monoclonal antibody targeting IL-1β which has demonstrated efficacy in the treatment of systemic onset juvenile idiopathic arthritis (So-JIA) (45). However, while anakinra demonstrates a degree of efficacy in systemic inflammation, it has proven ineffective for the treatment of IBD (46, 47). Therefore, it is evident that the role(s) of IL-1 family members in the GIT extend beyond the elicitation of inflammatory responses and these cytokines are also required for the maintenance of optimal barrier function. As outlined below, the role of the IL-1 family in GI homeostasis or IBD pathogenesis depends on factors including disease state, genetic predisposition due to gene polymorphisms, concomitant stimuli derived from the microbiota and the relative abundance of natural antagonists. Furthermore, both CD and UC are associated with different immunological profiles, with the former characterized by a robust T_H_1 response while the latter induces an immune signature more akin to a T_H_2-type response. Thus, when discussing the contributions of certain cytokines or other immunological factors in the context of IBD, it is important to note that their roles may differ not only between CD and UC but also depend on the stage of the disease. Herein we will discuss the roles of the IL-1 family members and their associated signaling pathways in the context of IBD and highlight the potential manipulation of this cytokine network for therapeutic purposes.

## IL-1α and IL-1β

IL-1, the first non-interferon cytokine to be discovered, consists of two different isoforms—IL-1α and IL-1β–both of which utilize the same IL-1 receptor (IL-1RI) and have similar biological signaling pathways and functions despite sharing only 26% sequence homology to one another (48). Upon cytokine binding to IL-1RI, hetero-dimerization of the receptor chain with the IL-1R accessory protein (IL-1RAcP) facilitates receptor complex formation, thereby providing a platform to which the adaptor protein MyD88 can bind and transduce signals to downstream kinases in a manner analogous to MyD88-dependent TLR signaling. As previously alluded to, a common feature of almost all IL-1 family members, with the exception of IL-1Ra, is a requirement for post-translational processing. This is true of both IL-1α and IL-1β, the precursors of which are cleaved by a variety of enzymes. However, while the pro-form of IL-1β is functionally inert and incapable of binding to IL-1RI, full-length IL-1α is bioactive (49, 50). Cleavage of pro-IL-1β is carried out by an enzyme first designated as IL-1β converting enzyme (ICE) and later renamed caspase-1, which forms part of a multi-protein complex known as the inflammasome (51, 52). In contrast, whereas processing of full-length IL-1α augments its bioactivity, this process occurs independently of caspase-1 (53). A seminal study by Afonina et al. has demonstrated that several inflammatory proteases, including calpain, elastase, granzyme B and mast cell chymase, are all capable of cleaving IL-1α and thus enhancing its affinity for the IL-1RI (54). In addition to this dichotomy in the activation states of their respective pro-forms, the two isoforms also differ in terms of their cellular localization. While IL-1α can typically be found in the cytosol both constitutively and following infection or cell damage, from where it can localize to the nucleus to modulate gene transcription in an IL-1RI-independent manner, IL-1β exhibits no intracellular functionality and must be actively secreted from the cell in response to stimuli (55). However, should the cell undergo inflammatory cell death, IL-1α is released into the extracellular environment and functions as an alarmin, signaling via IL-1RI expressing cells to initiate inflammation (56, 57). The bioactivity of this extracellular IL-1α can also be greatly potentiated by inflammatory proteases (54).

The first identified and to date best characterized endogenous regulator of IL-1 bioactivity is IL-1Ra, a natural antagonist of IL-1RI which is typically produced simultaneously with IL-1 as a means to restrict its signaling potential and prevent excessive stimulation of pro-inflammatory responses. The importance of this receptor antagonism is most evident in patients with a deficiency in the IL-1 receptor antagonist (DIRA), a condition which results in dysregulated IL-1 signaling culminating in severe auto-inflammatory responses characterized by the development of swollen joints and pustular rashes (58). Blockade of this aberrant IL-1 signaling can be achieved via the administration of recombinant IL-1Ra, Anakinra, resulting in a complete abrogation of inflammatory sequelae. Pre-clinically, mice deficient in IL-1Ra (*Il1rn*^−/−^) are highly susceptible to several autoimmune diseases including psoriasis, diabetes and encephalomyelitis and are known to spontaneously develop arthritis. Recently, Rogier et al. have identified that the severity of this latter condition has a strong microbial component and could be attributed to dysbiosis amongst the commensal microbiota intrinsic to *Il1rn*^−/−^ mice. Absence of IL-1Ra resulted in aberrant type-17 responses both at the intestinal mucosa and systemically and was transferable to wild-type mice via fecal transfer or co-habitation. However, this IL-17-mediated auto-inflammation could be significantly attenuated through the use of antibiotics targeting the IL-17-driving bacteria, thus indicating that in addition to directly antagonizing IL-1 bioactivity, IL-1Ra also plays a critical role in maintaining a homeostatic and balanced microbiota in the GIT (59).

In addition to IL-1Ra, the biological functions of IL-1 are further regulated by soluble and membrane-bound analogs of the IL-1R called IL-1RII. IL-1RII, whether expressed on the cell surface or in its soluble form (sIL-1RII), serves as a decoy receptor which is capable of binding to IL-1β with greater affinity than IL-1RI. Importantly, IL-1RII is the only receptor of the IL-1 family which lacks an intracellular TIR domain and is thus incapable of initiating an intracellular signaling cascade (60). Furthermore, it is bound by IL-1Ra with far lower affinity than IL-1RI and so does not interfere with the natural antagonistic properties of the former (61). IL-1RII also competes with IL-1RI for binding to the IL-1RAcP and in doing so limits positive signal transduction via IL-1 responsive receptors. Interestingly, although up-regulated by anti-inflammatory factors such as glucocorticoids, IL-1RII expression is reduced by pro-inflammatory stimuli including LPS and TNFα, suggesting that the onset of inflammation may alleviate the regulation of the IL-1 pathway by IL-1RII (62, 63). An additional inhibitory receptor of the IL-1 family is single immunoglobulin IL-1 receptor-related molecule (SIGIRR), otherwise known as TIR8, the extracellular domain of which can interact with IL-1RI, thereby preventing its dimerization with the IL-1RAcP. Furthermore, the intracellular TIR domain functions as a decoy for components of the IL-1 signaling pathway such as TRAF6 and IRAK1, thereby sequestering them to limit their participation in active IL-1 signaling via functional IL-1Rs (64). This receptor appears to be of particular importance in the GIT as both IECs and intestinal DCs from SIGIRR-deficient mice exhibit a skewed immunological profile characterized by a constitutive up-regulation of inflammatory genes and enhanced responsiveness to TLR ligands. Moreover, *Sigirr*^−/−^ mice were found to exhibit enhanced susceptibility to both intestinal inflammation and carcinogenesis in dextran sulfate sodium (DSS)-induced colitis and azoxymethane (AOM)/DSS colo-rectal cancer (CRC) models, respectively (65, 66). Interestingly, SIGIRR has more recently been shown to block T_H_17 cell proliferation by antagonizing IL-1 signaling and glycolytic metabolism, a finding which may have particular importance in the context of spontaneous colitis which develops in *Il10*^−/−^ mice and in which T_H_17 cells can mediate significant pathology (67). Thus, it would appear that the expression of this IL-1 family receptor by both hematopoietic and non-hematopoietic cells in the GIT is integral to the maintenance of immune homeostasis.

IL-1α in its precursor form, released from damaged IECs following onset of colitis, acts as a classical alarmin by initiating local inflammation. Interestingly, exogenous administration of anti-IL-1α antibodies, but not IL-1Ra, has been shown to ameliorate intestinal inflammation and increase repair and recovery of the epithelial barrier following DSS-induced colitis (68). This important finding points to dichotomous roles for IL-1α and IL-1β in the context of intestinal inflammation, with the former acting as a mediator of disease pathogenesis. More recently, the protection against chemically-induced colitis conferred by IL-1α deficiency has recently been linked to compositional alterations in the steady-state microbiota of *Il1a*^−/−^ mice and was shown to be abrogated following co-habitation of these animals with wild-type mice prior to the onset of colitis (69). Thus, it appears that in the context of IBD, IL-1α may contribute to disease pathogenesis in an indirect fashion by modulating the environmental niche occupied by commensal bacteria in the GIT.

While the role of IL-1α as a driver of inflammation has been reviewed elsewhere (70), the majority of work pertaining to IL-1 in the context of IBD has focused on IL-1β, levels of which are elevated in IBD (71–74). Mononuclear cells, predominantly macrophages of monocytic origin, residing within the LP are the primary source of these increased concentrations of IL-1 found in inflamed tissue biopsies during active IBD, with the extent to which these levels are elevated correlating with overall disease severity (75). Almost all cells at the intestinal mucosa express IL-1Rs and thus have the ability to respond to IL-1 stimulation. Therefore, deviations away from the concentrations of IL-1 found at steady-state has the potential to impact upon diverse populations of cells including those of myeloid, lymphoid and non-hematopoietic lineages which could significantly alter the immunological profile of the GIT. Indeed constitutive, intestinal macrophage-derived IL-1β signals produced in response to commensal stimuli has been deemed essential for the generation of homeostasis-promoting T_REGS_ under steady-state conditions in a recent study by Sonnenberg and colleagues. This response was found to be dependent on IL-1β-responsive ILC3s, which could drive the differentiation of T_REGS_ via their production of the proliferative cytokine IL-2. Ablation of IL-1 signaling specifically on ILC3s abrogated this induction of T_REGS_ and oral tolerance to dietary antigen. Interestingly, reduced production of IL-2 by ILC3s was found to be a feature of patient's with active CD, suggesting that this pathway may be of clinical importance and targetable (76).

In addition to IBD, elevated concentrations of IL-1β are also found in patients with colo-rectal cancer (CRC) and typically correlate with a poor prognosis. This is because in addition to its classical pro-inflammatory functions, IL-1β can also drive metastasis by promoting vascularization and cellular extravasation. Moreover, metastatic cancerous cells have been reported to express more IL-1RI than healthy bystander cells, suggesting that IL-1 can be utilized by these cells to facilitate further growth of the tumor (77). Despite this, therapeutic inhibition of IL-1 has so far proven ineffective for the treatment of IBD, suggestive of diverse roles for the IL-1 family in the maintenance of intestinal homeostasis and disease pathogenesis. Indeed in the aforementioned study by Bersudsky et al. neutralization of IL-1α, but not exogenous administration of rIL-1Ra or anti-IL-1β antibodies, ameliorated intestinal inflammation following DSS administration (68). Rather than simply quantifying circulating or mucosal concentrations of IL-1α or IL-1β in IBD patients to gather information pertaining to the stage or severity of disease flare-up, a more reliable and predictive readout has been described in which the ratio of IL-1Ra:IL-1 is measured. As reported by Casini-Raggi et al. an imbalance in the relative abundance of either of these compounds may be indicative of a perturbation of intestinal homeostasis and their work demonstrated that this ratio was shown to correlate strongly with disease severity amongst the cohort of IBD patients evaluated in their study (78). This observation is particularly noteworthy as it has previously been reported that in order to efficiently inhibit IL-1 signaling, IL-1Ra must be present in an approximate 100 fold excess compared to IL-1 (79). Furthermore, a simultaneous analysis of circulating and intestinal IL-1-associated factors may be more informative.

### IL-1 and Mucosal Antibody Secretion

In addition to its well established pro-inflammatory functions, IL-1β has more recently been shown to play an important, seemingly non-redundant role, in the differentiation of T_H_17 cells and activation of IL-17 secreting innate cells in the GIT under steady-state conditions (80, 81). This manifestation of IL-1 signaling was observed by Shaw et al. in a study in which they reported both a significant reduction in the number of intestinal T_H_17 cells as well as a decrease in local IL-17 production in the absence of IL-1R and/or MyD88 signaling (80). More recently, Basu et al. have provided further mechanistic insight into how IL-1 regulates the generation of T_H_17 cells *in vitro* and *in vivo*, a function which is linked to the dual role of transforming growth factor beta (TGF-β) in the differentiation of both T_H_17 and inducible T_REG_ cells (82, 83). Using the T_H_17 cell-inducing enteric pathogen *Citrobacter rodentium*, these studies demonstrated that IL-1 was necessary to overcome retinoic acid-mediated inhibition of T_H_17 cell differentiation by enhancing signal transducer and activator of transcription 3 (STAT3) activation, an effect facilitated via repression of its negative regulator, suppressor of cytokine signaling 3 (SOCS3). While phosphorylation of STAT5 in response to retinoic acid was not affected by concurrent IL-1 signaling, this sustained inhibition of SOCS3 enabled accessory T_H_17-driving cytokines, including IL-6, to induce further STAT3 phosphorylation which in turn increased T_H_17 cell frequency (84).

Since their discovery, the role of T_H_17 cells and their signature cytokine IL-17A in auto-inflammatory disease settings has been the subject of extensive research and in a number of cases, IL-1β has been identified as a critical factor responsible for driving IL-17-mediated disease pathogenesis. For example, T_H_17 cells have been identified as mediators of disease pathogenesis in experimental autoimmune encephalomyelitis (EAE)—the mouse model of multiple sclerosis (MS)—and blockade of IL-17 signaling results in a marked reduction in disease severity in this model. Moreover, *Il1r1*^−/−^ mice exhibit markedly reduced inflammation suggesting that the auto-reactive T_H_17 cells induced during EAE are dependent on IL-1 for their differentiation (41). More recent work has also implicated IL-1-responsive and IL-17-secreting γδ T cells in the pathogenesis of EAE, re-affirming this IL-1/IL-17 axis as a key propagator of inflammatory sequelae (85). Thus, it appears that blockade of IL-1 signaling and, by extension, the inflammasome from which much IL-1 is derived, might be prime targets for therapeutic strategies aimed at reversing the clinical symptoms of autoimmune inflammatory diseases characterized by hyper-active type 17 responses.

Ivanov et al. has shown that of 80–90% of IL-17 producing cells in the SI-LP are TCRαβ^+^ CD4^+^ T_H_17 cells, which require instruction via MyD88-dependent IL-1 signaling for their differentiation *in vivo* (86). Thus, it is plausible that inhibiting IL-1 signaling, or its production via inhibition of the inflammasome, may result in a significant reduction in the number and frequency of intestinal T_H_17 cells. While this may be beneficial in an acute inflammatory setting, the complete abrogation of IL-17 responses may have detrimental consequences for homeostatic processes which depend on intact IL-17 signaling. Such concerns are particularly pertinent when one considers the recently determined role for IL-17 in the maintenance of polymeric immunoglobulin receptor (pIgR) expression on mucosal epithelia (87, 88). This receptor, which is highly expressed on the basolateral membrane of IECs, facilitates the transport of dimeric immunoglobulin A and pentameric immunoglobulin M (dIgA and pIgM, respectively) across the epithelial barrier and into the lumen of the GIT and is therefore essential for intestinal homeostasis as demonstrated by studies in *Pigr*^−/−^ mice which exhibit enhanced susceptibility to DSS-induced colitis (89). Mice lacking the IL-17 receptor display comparable susceptibility to *Pigr*^−/−^ mice in this model owing to diminished epithelial pIgR expression and significantly reduced luminal IgA concentrations (87). Moreover, these animals harbor a dysbiotic microbiota characterized by an outgrowth of “colitogenic” segmented filamentous bacteria (SFB) which can closely adhere to the apical surface of IECs and exacerbate intestinal inflammation by driving excessive T_H_17 responses (90). Under steady-state conditions, these pathobionts are sequestered in the lumen where they are found to be bound by IgA as depicted in Figure 1. This coating of these microbes by endogenous antibodies is viewed as an essential process to remain tolerant to their presence and IgA exerts a profound influence over microbiota composition (91, 92). Interestingly, IL-1β itself has recently been shown to directly influence not only the transport, but also the production of IgA within the GIT in two independent studies. By developing a murine primary IEC system, Moon et al. demonstrated that IL-1β supported IgA transcytosis by directly regulating pIgR expression by IECs whereas Jung et al. identified eosinophil-derived IL-1β as a critical factor for optimal IgA production in the small intestine. Taken together, these studies therefore further highlight the importance of IL-1β as a regulator of homeostasis within the GIT (93, 94).

## IL-18

Initially referred to as interferon gamma inducing factor (IGIF), IL-18 was first identified as a potentiator of type 1 responses and in combination with IL-12 can stimulate T_H_1 responses and NK cell functionality (95–97). Like IL-1β, IL-18 is initially translated as a high molecular weight pro-form which is incapable of binding to its cognate receptor and thus must undergo post-translational cleavage. Also like IL-1β, the cleavage of pro-IL-18 occurs in a caspase-1-dependent manner upon assembly of the inflammasome, thereby yielding the mature cytokine which can then be secreted from the activated cell. Although caspase-1 is the only protease shown to definitively process pro-IL-18 into its bioactive form, the finding that *Casp1*^−/−^, but not *Casp8*^−/−^, macrophages are capable of secreting IL-18 upon stimulation suggests that caspase-8 may also play a role in its activation (98, 99). Furthermore, other proteases suggested to act in extracellular processing of pro-IL-1β, including mast cell chymase and granzyme B, have also been reported to cleave full length IL-18 (100, 101).

Similar to the antagonism of IL-1β by IL-1Ra, IL-18 is also regulated by an endogenous inhibitor called IL-18 binding protein (IL-18BP). However, unlike IL-1Ra which antagonizes the IL-1RI, IL-18BP binds directly to mature IL-18 with high affinity thereby preventing it from engaging the IL-18R on target cells. Crucially, IL-18BP does not bind to the pro-form of IL-18 and therefore only serves as a specific inhibitor for the active cytokine (102). Although expressed constitutively in certain tissues such as the spleen and GIT, IL-18BP levels are greatly enhanced in response to inflammatory stimuli including TNFα and IFNγ (103). Indeed work characterizing the expression of IL-18BP in human epithelial cells has demonstrated that these cells secrete elevated concentrations of IL-18BP in response to IFNγ (104, 105). Therefore, it appears that the production of this antagonist coincides with the onset of inflammation and in a manner analogous to IL-1Ra, serves to limit the signaling of its target cytokine to prevent excessive activation of the IL-18 pathway.

The constitutive expression of IL-18 by IECs suggests a role in the maintenance of GI homeostasis. Interestingly, enhanced production of IL-18, specifically by LP-resident macrophages, is a feature of CD (106, 107). While early pre-clinical studies identified IL-18 as a mediator of inflammation-induced pathology in colitis models, more recent work by a number of groups using various inflammasome-deficient mice has suggested that administration of recombinant IL-18 (rIL-18) can overcome such deficiencies and restore protection from chemically-induced colitis, with the majority of these studies concluding that this is attributed to enhanced epithelial regeneration and barrier repair in response to IL-18 (108–110). Furthermore, ILC3-derived IL-22 has been shown to drive pro-IL-18 expression by IECs, thus conferring protection from intestinal inflammation induced by the murine enteric pathogen *Citrobacter rodentium* (111). In addition to acting upon cells in the epithelial layer, IL-18 has also been shown to modulate effector CD4 responses in the GIT. A recent study by Harrison et al. reported that IEC-derived IL-18 was essential for ensuring balance between colonic T_H_17 and T_REG_ differentiation at steady-state and that this equilibrium was essential for the maintenance of homeostasis (112). Interestingly, in this context IL-18 acted as both an activatory and inhibitory factor, signaling via the IL-18R to promote the differentiation of Foxp3^+^ T_REGS_ while simultaneously directly antagonizing IL-1RI-dependent T_H_17 differentiation (112). However, this assumed barrier protective role for IL-18 in the GIT has been challenged in a recent study by Nowarski et al. in which they demonstrated that IL-18 interfered with goblet cell differentiation and maturation with detrimental consequences. They demonstrated that conditional deletion of *Il18r* in epithelial cells (*Il18r*^Δ*IEC*^) conferred protection against DSS-induced colitis and could reverse the inflammatory phenotype exhibited by mice deficient in the IL-18BP (113). However, these results are in contrast to a previous report by the same group in which they associated defects in the mucus barrier of *Nlrp6*^−/−^ mice and resulting susceptibility to both DSS- and *C. rodentium*-induced colitis with reduced levels of IL-18 (114, 115). In light of these conflicting data, at this stage it is difficult to determine the precise contribution of IL-18 in the context of IBD as the cytokine may play dichotomous roles depending on the stage of disease or its target cells. Furthermore, as is the case with IL-1Ra, IL-18BP levels are also increased in IBD and while the ratio of IL18:IL-18BP appears to correlate with the severity of inflammatory episodes in individuals with eczema and asthma, this has not been established in the context of IBD (116, 117). However, since GWAS studies have linked mutations in the IL-18 pathway with IBD susceptibility, it would appear that IL-18 is a key constituent of the host response to intestinal inflammation and therefore of interest therapeutically (118–120).

## IL-33

IL-33 is unique amongst the IL-1 family cytokines in that it preferentially induces type 2 immune responses (121). First discovered in humans in 2003 where it was found to be constitutively expressed in the nucleus of cells in barrier tissues throughout the body, IL-33 was further characterized by Schmitz et al. as a cytokine that induced T_H_2 cytokines and eosinophilia *in vivo* via binding to what was then the orphan cytokine receptor T1/ST2 (ST2) (121, 122). Whilst best known for its pathogenic role in asthma and allergy, its presence in the gut epithelium and its role as an “alarmin” released during tissue damage has highlighted it as a potential target in IBD.

IL-33 is constitutively expressed by non-haematopoietic cells in barrier tissues, including the gut mucosa (122–125). It is primarily expressed by epithelial and endothelial cells but expression also occurs in activated fibroblasts and myofibroblasts, which, in the gut, includes peri-cryptal fibroblasts (126). While mRNA expression of IL-33 has been widely reported in immune cells during inflammation, the functional consequence(s) of this is unknown and IL-33 remains primarily viewed as an epithelial-derived cytokine (123, 127, 128). Like other IL-1-family members, IL-33 is transcribed as a pro-form, referred to as full length-IL-33 (FL-IL-33), a 30 KDa protein containing a N-terminal chromatin-binding motif responsible and a C-terminal IL-1-like domain which mediates its cytokine activity (122, 123, 129). Akin to IL-1α, IL-33 lacks a signal sequence for secretion and owing to its chromatin-binding moiety, FL-IL-33 is found exclusively in the nucleus of viable cells, from where it may be capable of fulfilling a secondary role as a transcriptional repressor. Indeed its expression is associated with epithelial cell maturity and quiescence (129, 130). Interestingly, loss of the nuclear localization domain of IL-33 leads to ST2-dependent lethal inflammation in mice; suggesting firstly that IL-33 is a highly inflammatory cytokine, and secondly that its sequestration in the nucleus is a regulatory mechanism which limits its activity (131). Bound up in chromatin and with no described mechanism of active release, the presence of IL-33 in the extracellular space is thought to be dependent on passive release from dead or damaged cells (132). In this respect, IL-33 has been viewed as an “alarmin”, a signal of tissue damage (125, 133, 134). Diverse stimuli including bee venom, allergens such as *Alternaria* extract, the adjuvant alum as well as the physical damage associated with helminth infections have all been shown to induce release of IL-33 (123).

Unlike other IL-1-family members, with the exception of IL-1α, the pro-form of IL-33 is biologically active. However, while FL-IL-33 can bind and signal through ST2, a processed form of the cytokine consisting solely of the C-terminal IL-1-like domain, exhibits 10–30 times greater potency (135–137). This proteolytic cleavage of FL-IL-33 is achieved by many of the same enzymes responsible for the extracellular cleavage of IL-1α including neutrophil-derived cathepsin G and elastase, in addition to mast cell chymase, tryptase and granzyme B (136, 137). Thus, activity of IL-33 is greatest when it is released into an environment which already contains an inflammatory cell infiltrate.

In contrast to the processing of the pro-forms of IL-1β and IL-18 into the bioactive cytokines, cleavage of FL-IL-33 by caspases including caspase-1, −3 and −7 render it incapable of binding to ST2, thus limiting the inflammatory potential of apoptotic cells (133, 134, 138). The half-life of IL-33 is typically very short and the cytokine is often undetectable within hours of its release. It has been proposed that this rapid disappearance may be a result of natural oxidation of the cytokine in the extracellular space, leading to the formation of disulphide bridges and a conformational change that prevent binding to ST2 (139).

The receptor for IL-33, ST2, is constitutively expressed on a variety of cells of both myeloid and lymphoid origin including mast cells, T_H_2 cells, ILC2s and tissue-resident T_REGS_. In addition, although macrophages and eosinophils possess low basal levels of ST2, both can rapidly up-regulate its expression once activated (127, 132, 140, 141). The ST2 protein exists as two membrane bound splice variants ST2L and ST2V, and a soluble isoform (sST2) which lacks a transmembrane domain and is released from the cell surface (142). sST2, whose expression is controlled by activation of a secondary promoter site for ST2, is produced by immune cells during inflammation and acts as a decoy receptor, analogous to sIL-1RII, thereby limiting the biological activity of IL-33 (143).

Once bound by mature IL-33, ST2 recruits IL-1AcP to initiate signal transduction which follows classical IL-1 signaling and involves MyD88, TRAF6 and IRAK1/4 leading ultimately to activation of the NFκB and MAP-Kinase pathways (121, 123, 141, 144). The intracellular kinases downstream of ST2 appear to differ slightly depending on cell type, with NFκB activation being required for the production of pro-inflammatory cytokines such as IL-6 and TNF-α by mast cells whereas MAP-Kinase appears to be the dominant pathway engaged in T cells and ILC2s (127). Analogous to its role in IL-1RI signaling, SIGIRR is capable of inhibiting IL-33 signaling at this stage by competing with ST2 for binding to IL-1RAcP (145).

The canonical role of IL-33 is in the elicitation of type 2 immunity and it is integral to the innate response to certain helminth infections (140, 146–150). In addition, GWAS studies have proposed an association of certain mutations in IL-33 and ST2 with susceptibility to allergy and asthma (151).

IL-33 directly activates mast cells independently of IgE and is an important factor promoting the antigen-independent production of IL-5 and IL-13 by tissue-resident ILC2s and T_H_2 cells (140, 152). Here, the effects of IL-33 often synergize and overlap with those of other epithelial-derived type 2 cytokines including thymic stromal lymphopoietin (TSLP) and IL-25 (148). Recently, a role for IL-33 in the elicitation of regulatory immune responses has also been uncovered with the identification of a population of ST2-expressing tissue-resident T_REGS_, the suppressive function of which is enhanced by IL-33 (153). Furthermore, IL-33 release following tissue damage in mice has been shown to be required for the initiation of effective wound healing (154, 155). Thus, IL-33 may be best viewed as a highly-regulated cytokine which alerts the immune system to tissue damage but which also promotes the induction of responses that resolve inflammation.

As mentioned, IL-33, like IL-1α, is constitutively expressed by IECs and is released following tissue damage (142, 156–158). Exogenous IL-33 increases gut epithelial cell proliferation and mucus production (159). Moreover, there is some evidence that IL-33 signaling may affect gut motility via acting upon the enteric nervous system (160). It was reported that IL-33-deficent mice have a defect in IgA production and an altered microbiota (142, 160).

The fact that IL-33 promotes type 2 immune responses has implications for its potential differential role in CD vs. UC. While IL-33 is not associated with CD, which is typically characterized by T_H_1 and/or T_H_17-dominated inflammatory responses in the gut, it has been associated with UC in which a pronounced T_H_2 profile is normally observed (161). Indeed concentrations of IL-33 are increased in the colon and serum of UC patients, leading to its identification as a potential pharmacological target (158, 162, 163). Interestingly, IL-33 is increased during disease flare-ups and decreased following anti-TNF-α-induced remission (164). Furthermore, IL-33 was found to induce T_H_2-associated cytokine secretion from mesenteric lymph node-derived cells of UC patients (158). However, it remains to be determined whether IL-33, in the context of UC, functions as a read-out of tissue damage or direct mediator of disease pathogenesis.

The role of IL-33 in pre-clinical models of IBD is unclear, with various studies demonstrating both protective and pathologic roles for IL-33. In support of the latter, Sedholm et al. reported that levels of IL-33 were enhanced in the intestinal epithelium of both DSS- and 2,4,6-trinitrobenzene sulphonic acid (TNBS)-treated animals. In both models, recombinant IL-33 (rIL-33) exacerbated the severity of the inflammation whereas ST2-deficient animals were somewhat protected (163). Two further studies corroborated the protective phenotype observed in *Il1rl1*^−/−^ mice and the exacerbating effect of IL-33 in the DSS model, with one study showing a role for IL-4 in mediating the damaging effects of IL-33 (157, 165). The apparent dichotomy was reflected in a study by Sattler et al. where exogenous administration of rIL-33 exacerbated spontaneous colitis in *Il10*^−/−^ mice, while concomitantly driving the expansion of a population of IL-10-producing B cells in wild-type mice. Moreover, these IL-10^+^ B cells were shown to constrain colitis upon transfer into *Il10*^−/−^ recipients, thereby further demonstrating the duality of IL-33 (166). To complicate the picture further, Groß et al. showed a time-dependent effect of IL-33 on DSS-induced colitis, where IL-33 worsened acute disease but led to improved recovery if administered following removal of DSS from the drinking water (167).

In direct contrast to these findings, others have found a protective role for IL-33 in experimental colitis. Two independent studies demonstrated that exogenous IL-33 attenuated TNBS-induced colitis, protective phenotypes which were attributed to the induction of either T_H_2/T_REGS_ or M2 macrophages, respectively (168, 169). Similarly, Seo et al. reported a protective role for IL-33-activated regulatory macrophages in DSS-induced colitis using cell transfers (170). Furthermore, in a seminal study describing a protective role for IL-33 in colitis, Malik et al. demonstrated that *Il33*^−/−^ mice were more susceptible to DSS-induced tissue damage and colitis-associated tumorigenesis, phenotypes which were both due to elevated concentrations of IL-1α in the absence of IL-33 and which could be ameliorated via abolition of IL-1α signaling (160). Interestingly, the authors demonstrated that these susceptibility phenotypes had a strong microbial element and that *Il33*^−/−^ mice exhibited reduced concentrations of intestinal IgA and harbored a dysbiotic microbiota, characterized by an increased abundance of the colitogenic bacterium *Akkermansia muciniphilia*. Selective reduction of this bacterium using a specific antibiotic regimen or equilibration of the microbiota via co-habitation with wild-type animals successfully attenuated the exacerbated disease symptoms otherwise observed in *Il33*^−/−^ mice (160).

Some of the inconsistencies might in part be explained by the intermittent use of *Il1rl1*^−/−^ vs. *Il33*^−/−^ mice in several studies, the timing of administration of IL-33, or the use of different rIL-33 proteins, many of which differ in a unique way from FL-IL-33 with regard to their peptide sequence or post-translational modifications.

The interaction of IL-33 with T_REGS_ and T_H_17 cells in the gut is of particular interest given their reciprocal roles in colitis. Indeed the expression of ST2 is characteristic of colonic T_REGS_, the suppressive function of which can be enhanced by IL-33. Interestingly, IL-23, a key T_H_17-promoting cytokine, has been shown to antagonize this IL-33-mediated increase in T_REG_ activity (171). In addition to modulating mucosal T_H_17 responses indirectly via T_REGS_, recent work by Pascual-Reguant et al. identifying ST2 on the surface of intestinal T_H_17 cells has suggested that IL-33 may also act upon these cells directly to constrain their effector functions. Using a murine model of anti-CD3-induced colitis, they showed that ST2-expressing T_H_17 cells exhibited a reduced capacity for proliferation and secretion of IL-17A but enhanced IL-10 production in response to IL-33 (156). Furthermore, ligands of the epidermal growth factor receptor (EGFR), including amphiregulin, may be important in accentuating the effects of IL-33 in the gut. ST2 and EGFR form a signaling complex which is required to mediate the effect of IL-33 on T_H_2 cells (172). Considering EGFR ligands have been implicated in tissue regeneration in UC, their interplay with IL-33 in IBD may have a role in protection from disease (173). In this respect, Monticelli et al. have reported that amphiregulin, produced by ILC2 in response to IL-33, conferred protection against DSS-induced colitis by enhancing tissue repair (159).

It must be noted that the conflicting role of IL-33 as reported in pre-clinical models of IBD extends beyond the gut and IL-33 has been found to have both protective and exacerbating effects in systemic models of autoimmunity including EAE (174, 175). Thus, similar to other IL-1 members, IL-33 likely plays a dichotomous role in inflammatory disorders by initiating inflammation following its release from damaged cells while also inducing type 2 or regulatory responses that lead to resolution of inflammation and tissue repair.

## IL-36

Although initially described as an extension of the prototypical IL-1 family, within the last decade IL-36 cytokines have been designated as a distinct sub-family which signal through their own specific receptor—IL-36R (176–178). Similar to IL-1, the IL-36 subfamily consists of distinct isoforms—IL-36α, β and γ–and a natural antagonist—IL-36Ra (179). The three isoforms signal via the IL-36R complex which shares the accessory protein IL-1RAcP with other IL-1 family members and similarly contains an intracellular TIR domain, thus enabling its interaction with cytoplasmic MyD88 to propagate downstream intracellular signals (28, 180). Although these cytokines lack classical caspase cleavage sites, post-translational cleavage of IL-36α, -β, and -γ, as well as IL-36Ra, at the N-terminal has been shown to dramatically enhance their potency *in vitro* (181). Furthermore, the lack of signal sequence suggests that these cytokines are not actively secreted from the endoplasmic reticulum and neutrophil-derived proteases implicated in extracellular cleavage of full length IL-1α and IL-1β, namely cathepsin G, elastase and proteinase-3, have been proposed as activators of the IL-36 sub-family members (182, 183). Thus, in a manner analogous to IL-1α and IL-33, the release of these cytokines may be a consequence of inflammatory cell death enabling them to acts as alarmins and initiate inflammatory cascades by binding to IL-36R-expressing cells. Due to this potential to accelerate inflammatory responses, IL-36 cytokines, like other IL-1 family members, are tightly regulated. Their activity is primarily limited by IL36Ra, which shares 52% sequence homology with and performs an analogous function to IL-1Ra. As previously mentioned, patients with IL-1Ra deficiencies develop systemic auto-inflammation characterized by swollen joints and pustular rashes (58). A similar disorder results from a rare inactivating mutation in the gene encoding IL-36Ra, *IL36RN*, and is manifested as a severe inflammation of the skin referred to as generalized pustular psoriasis (GPP) (184). Additionally, further intrinsic regulation comes in the form of IL-38, another IL-1 family member with a high degree of homology with both IL-36Ra (43%) and IL-1Ra (41%) and which is capable of binding to the IL-36R (185).

IL-36 cytokines are expressed either constitutively or in response to inflammatory stimuli by human and mouse cells including DCs, monocytes/macrophages, T cells and epithelial cells. IL-36R-expressing DCs can in turn respond to IL-36 by up-regulating prototypical maturation markers and cytokine production in a manner analogous to TLR stimulation (186). Thus, in this context, rather than function as a typical IL-1 family member, IL-36 primes DCs for subsequent modulation of effector responses. Indeed IL-36β has been shown to synergize with IL-12 to preferentially drive the expansion and activation of T_H_1 cells from naïve precursors (187, 188). Two recent, independent studies have indicated that levels of IL36α and IL-36γ are up-regulated in the colons of both IBD patients and mice in which intestinal inflammation is induced by DSS (189, 190). In the former of these two studies, Russell et al. demonstrated that *Il1rl2*^−/−^ mice were significantly less susceptible to both DSS- and *C.rodentium*-induced colitis, phenotypes which the authors attributed to a skewed T helper cell profile in the GIT of these animals. However, these results are in contrast to a study by Medina-Contreras et al. in which *Il1rl2*^−/−^ mice exhibited impaired resolution of inflammatory lesions and sub-optimal wound healing compared to littermate controls (191). Of note, the elevated levels of IL-36 cytokines observed in this study were not observed in germ free mice, leading the authors to postulate a possible role for the microbiota in intestinal IL-36 signaling. Scheibe et al. recently reported that treatment with IL-36 accelerated colonic wound healing by stimulating IEC proliferation and that this required MyD88 signaling. Importantly, by generating bone marrow chimeras, the authors demonstrated that IL-36R in the hematopoietic compartment was dispensable for this improved healing suggesting that the expression of the IL-36R by non-hematopoietic IECs or epithelial progenitors likely regulates this process (190). However, as is the case with several of the other IL-1 family members, further studies will be required to fully resolve the potential role of this subfamily of cytokines in IBD. In particular, in-depth characterization of the composition of the respective microbiotas of *Il1rl2*^−/−^ and *Il1rl2*^+/+^ mice in parallel will help to determine whether dysbiosis, as suggested by Medina-Contreras et al. may underlie some of the phenotypes reported in these animals. Furthermore, although Scheibe et al. identified the expression of IL-36R in the non-hematopoietic compartment as an essential component of the colonic wound healing process, more in-depth studies using conditional knock-out animals will be necessary for elucidating the cell-specific effects of these cytokines. Finally, epidemiological studies investigating potential links between the *IL36RN* mutation or the ratio of IL36Ra/IL38:IL-36 and susceptibility to IBD may identify one or more of these factors as candidate biomarkers for the disease.

## IL-37

IL-37 is an anti-inflammatory member of the IL-1 cytokine family, production of which is increased in a number of inflammatory conditions including psoriasis, RA and IBD. In inflamed tissue sections from UC and CD patients, IL-37 concentrations were reported to be enhanced, with follow-up *in vitro* assays revealing that this increased production was driven by TNFα as part of an auto-inhibitory feedback loop to limit TNFα-induced inflammatory responses (192). In this regard, IL-37 may perform a similar function to IL-1Ra and IL-18BP, the expression of which is also up-regulated during active IBD. In contrast, in patients with microscopic colitis, *IL37* gene expression was found to be reduced compared to healthy individuals with the former also exhibiting elevated levels of chemokines driving cellular infiltration into the intestinal LP (193, 194). Although five isoforms of IL-37 exist in humans, IL-37a-e, with IL-37b being the best characterized and most studied to date, there is no murine homolog, hence the limited literature pertaining to the role of this cytokine in inflammatory conditions. However, studies using transgenic mice expressing human IL-37b have demonstrated that these mice are more resistant to DSS-induced colitis (195, 196). The literature regarding IL-37 processing and signaling is similarly scarce, however a putative caspase-1 cleavage site has been reported which is conserved across four of the isoforms (197, 198). Similar to IL-1α and IL-33, it appears that IL-37 is also capable of exerting its biological effects both intra- and extra-cellularly. In the former scenario IL-37 translocates to the nucleus from where it can inhibit pro-inflammatory gene transcription whereas extracellular IL-37 engages IL-18Rα in association with SIGIRR to initiate an anti-inflammatory cascade (198–203). The ability of IL-37 to translocate to the nucleus appears to be caspase-1-dependent and is lost in cells lacking the inflammasome components ASC or NLRP3 (199). While the administration of recombinant IL-37 or a mimetic as an anti-inflammatory therapeutic may be a promising strategy for the treatment of auto-inflammatory conditions, further work will be required to identify the precise role of each of the IL-37 isoforms in the context of both inflammation and homeostasis and epidemiological studies might reveal whether any mutations or SNPs exist which could link alterations in this cytokine to disease susceptibility or severity.

## IL-38

In humans, the gene encoding IL-38 (*IL1F10*) is located within the IL-1 gene cluster on chromosome 2p13, adjacent to the genes encoding IL-1Ra and IL-36Ra (204). Indeed, as already alluded to, the IL-38 protein shares remarkable sequence and structural homology with these natural IL-1 family antagonists-−41% homologous to IL-1Ra and 43% to IL-36Ra. Perhaps unsurprisingly, IL-38 is therefore a regulatory cytokine capable of binding to several receptors and modulating the downstream function(s) of multiple pro-inflammatory cytokines, in particular those associated with a T_H_17 response. Pro-inflammatory cytokine secretion following *in vitro* stimulation of peripheral blood mononuclear cells (PBMCs) and macrophages, with TLR agonists was found to be markedly reduced when IL-38 was present. Additionally, production of the chemo-attractant IL-8 following treatment of PBMCs with IL-36γ was found to be abrogated in the presence of IL-38 to a similar extent as IL-36Ra (205). Thus, it appears that the function of IL-38 is analogous to that of other natural IL-1 family antagonists and similar to these molecules, it has been reported that IL-38 concentrations are elevated in patients afflicted by certain inflammatory conditions including RA, psoriasis, systemic lupus erythematosus (SLE), chronic obstructive pulmonary disease (COPD) and IBD. However, while many of these observations are based on serum cytokine concentrations, such read-outs are not always indicative of the response at distant sites and do not always correlate with disease severity. Indeed despite elevated IL-38 levels in the serum of patients with psoriasis, Palomo et al. have recently reported that IL-38-deficiency does not affect the severity of skin inflammation in a murine psoriasis model. Using an imiquimod-induced psoriasis model, they demonstrated that local *Il36rn*, but not *Il38* levels, were increased in the inflamed skin following topical application of Aldara™ and disease severity and progression was comparable between *Il1f10*^−/−^ mice and wild-type littermate controls (206). In contrast, *Il36rn*^−/−^ mice exhibited exacerbated inflammatory sequelae leading to the author's conclusion that in this model at least, IL-38 was dispensable for regulating local inflammatory responses in the skin and could not compensate for a lack of IL-36Ra in *Il36rn*^−/−^ mice. However, a recent study by Han et al. demonstrated that IL-38 could indeed ameliorate imiquimod-induced skin inflammation by limiting IL-17 production from γδ T cells. Here the authors showed that *Il1f10*^−/−^ mice exhibited prolonged, type-17-mediated skin inflammation, a phenotype which could be reversed via exogenous administration of recombinant human IL-38 (207).

Enhanced *Il38* levels have been reported in the colon of DSS-treated mice and also in samples obtained from CD patients where *IL38* levels were higher in inflamed colonic biopsies compared to non-inflamed tissue (208). Further studies will be necessary to determine whether IL-38 deficiency or blockade of IL-38 might result in unrestrained effector T cell responses and exacerbated intestinal inflammation. Moreover, given its role at limiting the production of T_H_17 differentiation factors, together with the recently uncovered role for T_H_17 cells and their signature cytokine IL-17A in the maintenance of intestinal homeostasis and composition of the microbiota, whether *Il1f10*^−/−^ mice exhibit microbial dysbiosis has yet to be established and warrants further investigation (87, 90).

## Inflammasomes in IBD

In light of the counterintuitive results obtained from studies investigating the inhibition of IL-1 signaling as a potential treatment for IBD, what has become strikingly apparent is that the inflammatory processes and markers associated with pro-inflammatory conditions in the periphery do not always hold true within the GIT. As previously alluded to, caspase-mediated cleavage of the pro-forms of IL-1β and IL-18 typically occurs as part of a larger complex termed the inflammasome. These high molecular weight, multi-protein complexes serve as platforms for the recruitment and activation of pro-inflammatory caspases, such as caspase-1 and−5 (209, 210). As a result, they are critical components of the inflammatory response to noxious stimuli as they bridge the initial sensing of PAMPS or DAMPs and subsequent stimulation of innate immune mechanisms with the downstream modulation of stimulus-specific adaptive immunity. Although several such complexes exist, each inflammasome is structured around a core set of elements including a sensor protein, an adaptor molecule and an inflammatory caspase. While inflammasomes can be broadly sub-divided into two distinct, but functionally similar groups on the basis of their sensor proteins—those of the Nod-like receptor (NLR) and PYHIN families—we will focus on the former for the purpose of this review due to the extensive literature pertaining to their role(s) in IBD.

**Figure 4:**
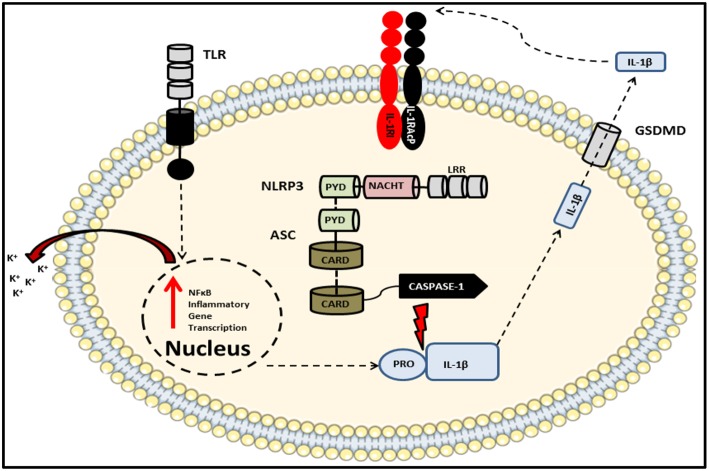
Schematic of the NLRP3 inflammasome complex. The prototypical inflammasome-forming NLR consisting of the sensory component NLRP3 bound via PRYIN domain interactions with the adaptor protein ASC, which in turn associates with caspase-1 via their respective CARD domains. An initial stimulus, such as a TLR ligand, primes the inflammasome via the ligand sensing LRR domain, thereby initiating oligomerization of NACHT domains and activation of the inflammasome which results in the production of cytokines including pro-IL-1β and pro-IL-18. A subsequent second signal, such as potassium ion efflux, induces the autocatalysis and activation of caspase-1. This autocatalytic event enables the enzyme to cleave its substrates which include both pro-IL-1β and pro-IL-18 as well as the pore-forming protein gasdermin D. Once released into the extracellular space, these bioactive cytokines can go on to signal in an auto- or paracrine manner.

The NLR family includes NLRP1, NLRP2, NLRP3, NLRP5, NLRP6, NLRP7, NLRC4, NLRP12, NLRP14, NOD1, NOD2, and absent in melanoma 2 (AIM2) and plays essential roles in pathogen recognition, the maintenance of homeostasis and embryonic development (211, 212). The impetus for studying the NLR family in the context of IBD was the identification of SNPs within the peptidoglycan-sensing *NOD2* gene which have been associated with enhanced susceptibility to CD (213, 214). Subsequent genome-wide association studies (GWAS) have identified several other mutations in NLR family members as risk factors correlating with IBD susceptibility. One of these members, NLRP3, has been the focus of the majority of inflammasome-based research and is therefore the best characterized of the NLR complexes identified to date. Recently however, there is accumulating evidence suggesting important roles for other NLR family members, in particular NLRP6, in the maintenance of intestinal homeostasis. Indeed the consequences of deficiencies in various inflammasome components across a diverse range of chemically- and pathogen-induced colitis models has been extensively reviewed elsewhere (215). Of the aforementioned NLRs, AIM2 as well as NLRP1, NLRP3, NLRP6, and NLRC4 are involved in the formation of inflammasomes. Although they differ in terms of their pattern of expression and the stimuli to which they respond, structurally they are similar and consist of a specific NLR, the apoptosis-associated speck-like protein containing a CARD (ASC) adaptor protein and an inflammatory caspase, typically caspase-1. Canonical inflammasome activation is primed by engagement of an innate receptor such as a TLR on the cell surface and fully initiated upon receipt of a secondary signal which can be in the form of an exogenous stimulus or an intracellular event such as the efflux of potassium as depicted in Figure 4, resulting in the association of ASC with the NLR via their respective pyrin domains. This interaction triggers the formation of the ASC speck, a multimeric complex made-up of several ASC dimers which acts as a scaffold to which caspase-1 monomers can bind via their CARD domains. This interaction of caspase-1 with ASC induces an auto-cleavage event in the enzyme which converts it from its catalytically inactive, zymogen form, into a proteolytically active enzyme capable of cleaving pro-IL-1β and pro-IL-18 (209). In the case of IL-1β, caspase-1-mediated cleavage of the 31 kD pro-form yields a 17 kD bioactive cytokine which can then be actively secreted and bind to its cognate receptor on target cells.

In addition to its classical role in the functional maturation of pro-IL-1β and pro-IL-18, assembly of the inflammasome in this way can also trigger a form of cell death known as pyroptosis which is characterized by the activation of inflammatory caspases such as caspase-1,−5, and−11 (216–218). This leads to cleavage of gasdermin D (GSDMD) which, like pro-IL-1β and pro-IL-18, contains consensus caspase cleavage sites. Cleaved GSDMD, upon trafficking to the cell membrane induces cell death by forming oligomeric pores in the lipid bilayer (219). The resulting release of intracellular contents into the extracellular matrix via these pores is what distinguishes pyroptosis from quiescent apoptotic cell death, in which dying cells release membranous blebs containing their contents which are subsequently engulfed by nearby phagocytes. It is however important to highlight that unlike spontaneous cell death via necrosis, pyroptosis is programmed and thus restricted to the activated cell. Indeed pyroptotic cell death is an efficient host defense mechanism as it abolishes the intracellular niche required by certain pathogens to facilitate their replication (220).

However, an important caveat to these mechanistic studies is that for the most part they have been carried out *in vitro* using specific protocols whereby signal one is provided in the form of a TLR agonist such as LPS, followed at a defined amount of time later by a second stimulus that induces the release of mature cytokines. In reality, the *in vivo* scenario is likely to be very different to this controlled experimental setting as both signals are likely to be provided simultaneously and the concentrations of each stimulus is likely to differ greatly. This is particularly true of the GIT where perturbations in homeostatic mechanisms expose inflammasome-expressing IECs and LP-resident myeloid cells to a plethora of PAMPs derived from commensal microbes. Indeed disruption of the microbiota following treatment with broad-spectrum oral antibiotics has recently been shown to enhance the production of pro-inflammatory cytokines by intestinal macrophages in response to TLR ligation *ex vivo* (221). The overall composition of the microbiota itself is another confounding variable in animal studies which is likely to differ greatly depending on host genotype, supplier, husbandry practices and the environment in which the animals are housed. Certain commensal populations can have a profound influence on disease severity in DSS-induced colitis models and can exert host protective or deleterious effects by modulating the inflammatory responses elicited (86, 222–224). Indeed the significance of the microbiota as a key factor involved in colitis severity has been highlighted in a recent study by by Britton et al. in which colonization of gnotobiotic mice with fecal microbiotas from IBD patients significantly increased disease severity in a T cell transfer model of colitis compared with mice colonized with microbiotas from healthy control subjects. This exacerbated inflammatory disease phenotype correlated with marked alterations in the CD4+ T cell compartment of the GIT in mice receiving an IBD-associated or healthy microbiota (225).

Despite the clearly defined role of the NLR inflammasomes in the maturation of inflammatory cytokines and initiation of inflammatory cell death by pyroptosis, several studies modeling inflammasome deficiency through the use of *Pycard*^−/−^ mice have shown that these animals are intrinsically hyper-susceptible to chemically-induced colitis and exhibit several symptoms characteristic of severe intestinal inflammation including enhanced weight loss, diarrhea, rectal bleeding, colonic shortening and mortality. Furthermore, barrier integrity is compromised in these mice and they have been shown to exhibit elevated levels of serum endotoxin as well as bacterial translocation compared to wild-type mice (109). Importantly, *Pycard*^−/−^ mice are deficient in all inflammasome complexes and the severity of the inflammation they exhibit is greater than that of strains lacking individual NLRs. Thus, despite functioning as drivers of inflammatory responses in response to diverse stimuli, it appears that the NLR inflammasomes confer a degree of natural protection against the onset of gastro-intestinal inflammation. One of the major co-morbidities associated with IBD is the enhanced risk of developing gastro-intestinal tumors knowns as colitis-associated cancer (226). Interestingly, long before the characterization of the inflammasome and description of caspase-dependent pyroptosis, ASC deficiency had been associated with a poor prognosis in both human breast and gastric cancers (227). Furthermore, more recent studies have demonstrated that *Pycard*^−/−^ mice also display enhanced tumor burden and metastasis compared to wild-types in a murine CRC model which involves exposure to AOM prior to DSS administration (228). Similar results were reported on *Casp1*^−/−^ mice in both DSS-colitis and AOM-DSS CRC models (110, 228–230). Despite, the evidence pointing to a protective role for inflammasomes in IBD, there are a number of caveats which must be considered before definitive conclusions can be drawn. Firstly, while the majority of the aforementioned studies have utilized the DSS-colitis model to study acute intestinal injury, the same results are not seen in models of spontaneous colitis in *Il10*^−/−^ mice, which develop intestinal inflammation characterized by aberrant T_H_17 responses, resulting in significantly elevated colonic concentrations of IL-17. Intriguingly, this phenotype can be reversed and the inflammation resolved by inhibiting IL-1 signaling or caspase-1 (231). Indeed, IL-1β, produced in response to microbial stimuli derived from commensal bacteria, plays a non-redundant role in the differentiation of homeostasis-promoting T_H_17 cells in the GIT (80). Thus, although protective in chemically-induced colitis models, it appears that IL-1 exacerbates T_H_17-mediated intestinal inflammation. Such findings are particularly interesting when one considers the recent description of inflammasome- and IL-1RI-expressing T_H_17 cells in the aforementioned study by Martin et al. These cells were found to be capable of self-propagation through autocrine IL-1 signaling and drove significant pathology in EAE (232). However, the existence of such cells within the GIT has yet to be reported.

A further layer of complexity stems from the recent discovery that the *Casp1*^−/−^ mice used in several of the early seminal studies investigating the role of the inflammasome in IBD are also deficient in caspase-11 (*Casp11*^−/−^), the murine homolog of human caspases−4 and−5 (233). Subsequent studies have also reported a protective role for caspase-11 in DSS-colitis using *Casp11*^−/−^ mice which exhibit exacerbated barrier destruction and increased intestinal permeability despite retaining fully functional caspase-1 (108, 234). Rather than full ablation of IL-1β and IL-18 as occurs in *Pycard*^−/−^ and *Casp1*^−/−^ mice, *Casp11*^−/−^ mice merely have reduced concentrations of these cytokines and exogenous administration of recombinant IL-18 restores protection to wild-type levels. In line with previous results outlining protective roles for the inflammasome in CRC, caspases−4 and−5 have also been reported to be up-regulated in IBD patients (235). Indeed a recent study has also identified caspase-11 as protective in the AOM-DSS model. In their study, Flood et al. reported heightened susceptibility and exacerbated tumor growth in *Casp11*^−/−^ mice compared to their littermate controls. The enhanced tumor burden was associated with reduced levels of both IL-1β and IL-18 which resulted in reduced STAT1 signaling and sub-optimal anti-tumor type I immunity (236). Given that IL-1 cytokines derived from activated inflammasomes evidently play a fundamental role in determining the severity of various gastro-intestinal disorders, consideration must also be given to the composition of the commensal microbiota in these mouse strains. For example, Demon et al. reported a marked reduction in the relative abundance of *Prevotellaceae* in their *Casp11*^−/−^ mice, levels of which were normalized following co-habitation with wild-type mice but did not affect overall disease severity (234). Additionally, both *Pycard*^−/−^ and *Casp1*^−/−^ mice had also previously been reported to possess alterations in microbiota composition compared to wild-types (115). Due to these confounding variables, it is difficult to attribute a precise role to caspase-1 in the context of inflammasome-mediated protection from intestinal inflammation. However, a recent study by Flavell et al. has challenged the accepted dogma and provided evidence for a pro-inflammatory and pro-tumorigenic role for caspase-1 in the GIT. By generating mice deficient in either caspase-1 or caspase-11 only, the authors demonstrated that the expression of caspase-1 within non-hematopoietic cells was responsible for exacerbating chemically-induced intestinal inflammation and tumorigenesis independently of the microbiota or caspase-11 (237). While these findings may help to explain why specific blockade of caspase-1 had previously been reported to ameliorate inflammatory symptoms in murine spontaneous colitis models, the exact functions of specific NLR inflammasomes in IBD remains incompletely resolved.

Further confusion has arisen recently regarding the role of the NLRP6 inflammasome in the GIT. Initial work by Elinav et al. reported a protective role for this sensor in experimental colitis models and identifying a dysbiotic microbiota intrinsic to *Nlrp6*^−/−^ mice characterized by an expansion of *Prevotellaceae* (114, 115). However, a recent study by Mamantopoulus et al. suggested these reported phenotypes to be artifacts resulting from so-called “legacy” effects pertaining to husbandry procedures. They demonstrated that in contrast to earlier reports, neither NLRP6 nor the adaptor protein ASC exerted influence on the make-up of the host's microbiota, thus highlighting the importance of properly controlled experimental protocols when investigating the impact of specific immunological factors on microbial ecology (238, 239). Contrasting phenotypes have also previously been reported from DSS-induced colitis studies in *Nlrp3*^−/−^ mice. While early work by Bauer et al. reported a pathologic role for NLRP3, several subsequent studies have intimated that this inflammasome sensor is in fact protective in this model (110, 240). Recently, Macia et al. have shown that dietary fiber induces non-hematopoietic activation of the NLRP3, but not NLRP6, inflammasome resulting in enhanced caspase-1 activity, IL-18 secretion and improved disease outcome while an activating mutation in *Nlrp3*- *Nlrp3*^R258W^ - was associated with increased resistance to colitis and CRC due to inflammasome-IL-1β-dependent remodeling of the microbiota (241, 242). In addition to these well characterized complexes, the less studied sensor NLRP1 has also been shown to affect the microbiota (243). However, in contrast to the assumed barrier-protective role for NLRP3, overt NLRP1 signaling was found to exacerbate inflammatory sequelae in response to DSS. Moreover, the expression of *NLRP1* was found to be increased in inflamed colonic biopsies from UC patients, suggesting a primarily pathologic role for this sensor (243).

In light of these and other reports, it has become abundantly clear that when discussing the contribution(s) of inflammasomes and IL-1 family members in the pathogenesis of IBD, the impact of the indigenous microbiota cannot be overlooked and has played a somewhat under-appreciated role in determining disease severity in earlier work. While recent studies have begun to shed light on this issue in an effort to explain the variable phenotypes reported to date, previous work on the role of the NLRP3 inflammasome and IL-1 itself may need to be re-visited. Furthermore, the implementation of standardized practices for studies related to inflammasome-deficiency and the microbiota in IBD has been championed by several recent review articles in an effort to alleviate some potential sources of inter-study variance (244–246).

## Perspectives

The use of monoclonal antibody-based therapies to specifically target and inhibit pro-inflammatory cytokines has been a highly efficacious and beneficial intervention for patients with chronic inflammatory disorders. Indeed several cytokine-targeted therapies, including those targeting the IL-1 and IL-17A pathways, are used clinically to treat conditions such as RA, MS and psoriasis. However, it has frequently been observed that these highly specific and efficacious treatments do not ameliorate symptoms of IBD and in many instances have led to an exacerbation of the disease symptoms. While the exact mechanisms underlying these failures have yet to be fully elucidated, it is highly plausible that such interventions could interfere with normal physiological processes, such as the active transport of secretory antibodies at the mucosae, in which such cytokines play integral roles. Moreover, the impact of prolonged or repeated use of such therapeutics on adaptive immunity or the composition of the commensal microbiota has not been investigated for the most part. Indeed, it is only due to the improvements of next generation sequencing techniques that the influence of the microbiota on IBD and other auto-inflammatory conditions has come to be fully appreciated. Thus, although IL-1 family cytokines remain attractive targets for IBD therapeutics, in order to fully resolve their role in IBD, additional studies utilizing cell-type-specific conditional knockout animals in which standardized protocols to limit the influence of inter-strain variances in the commensal microbiota are implemented will be required.

## Author Contributions

CM and CF drafted the manuscript following initial discussions with EL and prepared the figures and tables. EL edited the manuscript and provided input on content, figures and perspectives.

### Conflict of Interest Statement

The authors declare that the research was conducted in the absence of any commercial or financial relationships that could be construed as a potential conflict of interest.
